# The impact of E-cigarette vaping and vapour constituents on bone health

**DOI:** 10.1186/s12950-021-00283-7

**Published:** 2021-05-05

**Authors:** Thomas Nicholson, Aaron Scott, Matthew Newton Ede, Simon W. Jones

**Affiliations:** 1 Institute of Inflammation and Ageing, MRC-ARUK Centre for Musculoskeletal Ageing Research, University of Birmingham, Birmingham, B15 2TT UK; 2 The Royal Orthopaedic Hospital, Birmingham, B31 2AP UK

**Keywords:** E-cigarette, Vaping, Osteoblasts, Osteoclasts, Nicotine

## Abstract

**Background:**

In contrast to cigarettes, electronic cigarette use (E-cigarettes) has grown substantially over the last decade. This is due to their promotion as both a safer alternative to cigarettes and as an aide to stop smoking. Critically, upon E-cigarette use, the user may be exposed to high doses of nicotine in addition to other compounds including flavouring chemicals, metal particulates and carbonyl compounds, particularly in highly vascularised tissues such as bone. However, there has been limited investigation into the impact of E-cigarette usage on bone physiology, particularly over extended time periods and there are no clinical recommendations regarding E-cigarette usage in relation to orthopaedic surgery. This literature review draws together data from studies that have investigated the impact of E-cigarette vapour and its major constituents on bone, detailing the models utilised and the relevant mechanistic and functional results.

**Main body:**

Currently there is a lack of studies both in vivo and in vitro that have utilised E-cigarette vapour, necessary to account for changes in chemical composition of E-cigarette liquids upon vaping. There is however evidence that human bone and bone cells express nicotine receptors and exposure of both osteoblasts and osteoclasts to nicotine, in high concentrations may reduce their viability and impair function. Similarly, it appears that aldehydes and flavouring chemicals may also negatively impact osteoblast viability and their ability to form bone. However, such functional findings are predominantly the result of studies utilising bone cell lines such as MG-63 or Saos-2 cells, with limited use of human osteoblasts or osteoclasts. Additionally, there is limited consideration for a possible impact on mesenchymal stem cells, which can also play an import role in bone repair.

**Conclusion:**

Understanding the function and mechanism of action of the various components of E-cigarette vapour in mediating human bone cell function, in addition to long term studies to determine the potential harm of chronic E-cigarette use on human bone will be important to inform users of potential risks, particularly regarding bone healing following orthopaedic surgery and injury.

## Background

**Fig. 1:**
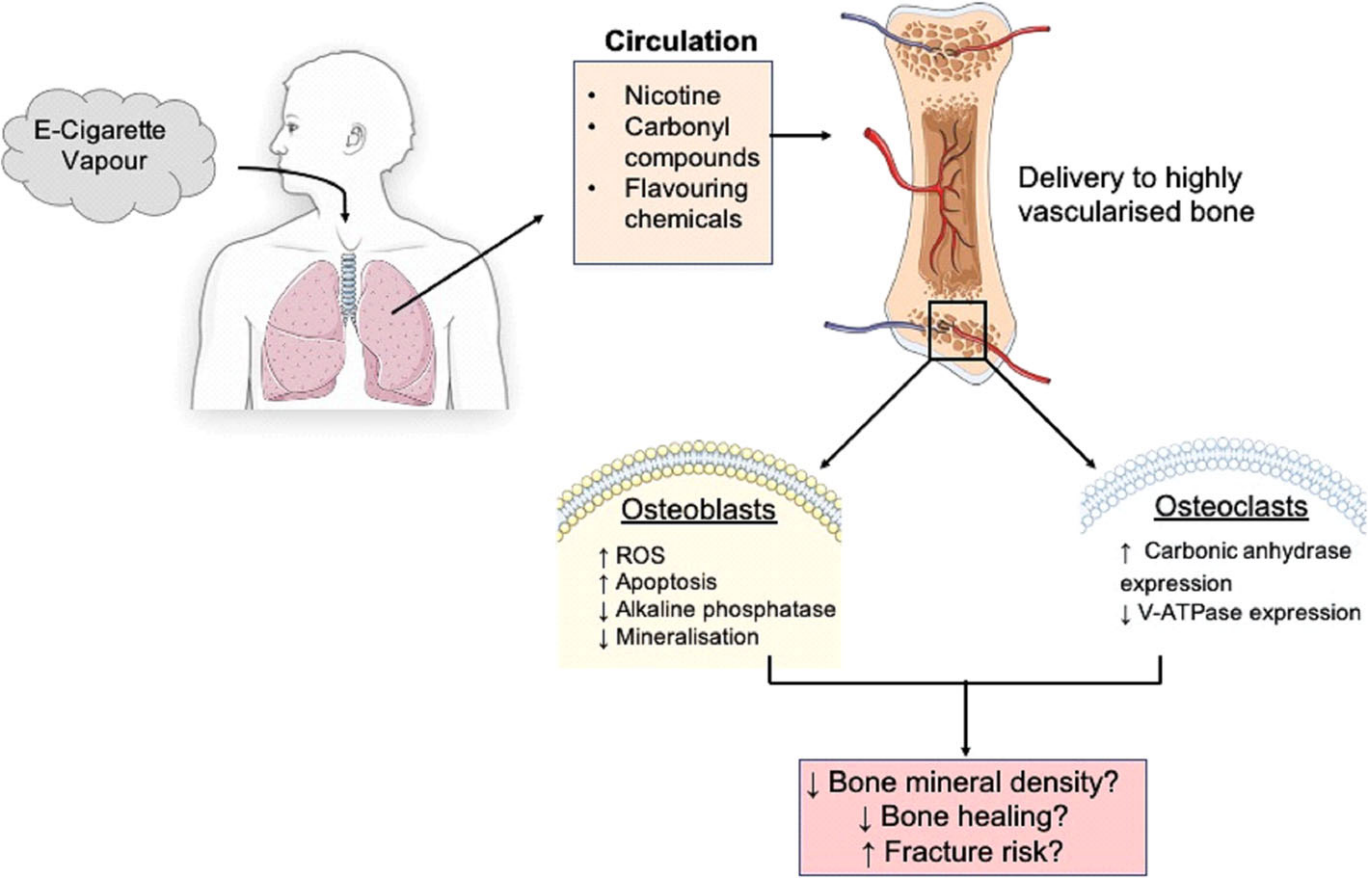
A potential mechanism to highlight how E-cigarette usage may impact bone

Multiple meta-analyses have reported that a history of cigarette smoking is significantly associated with reduced bone mineral density (BMD), increased risk of fracture and reduced fracture healing in comparison to non-smokers of the same age, sex and body mass index (BMI) [1–4]. It is also apparent that such smoking-associated effects are cumulative, demonstrating a positive correlation with pack year history [2, 3, 5]. Furthermore, fracture risk in smoking cohorts is greater than in non-smokers when corrected for BMD, indicating that smoking may directly impact bone architecture and quality [6]. Indeed, a decrease in trabecular bone mass and increased trabecular separation has been reported in older smokers [7], while in younger individuals smoking is associated with a reduction in trabecular bone volume, independent of age, BMI, activity level and calcium intake [8]. Smoking is also independently associated with increased post-surgery complications such as infection and aseptic loosening following arthroplasty [9–12]. In light of such data, patients are advised not to smoke cigarettes for a minimum of 4 weeks prior to orthopaedic surgery and continue to abstain post-surgery, in order to minimise complications, facilitate maximal bone healing and reduce aseptic loosening [13, 14]. While cigarette consumption has declined over the past decade, the use of electronic-cigarettes (E-cigarettes) or vaping, has risen dramatically, partly due to being regarded as a safer alternative to smoking [15–17]. Indeed, Public Health England guidance suggests E-cigarettes are 95% safer than cigarettes, fuelling public perception of negligible risk. Additionally, the use of E-cigarettes is reportedly twice as effective as nicotine replacement therapies in facilitating smoking cessation [18]. Therefore patients advised by health care professionals to quit smoking, are more likely to turn to E-cigarettes as a cessation aide [17]. Increased use of E-cigarettes will undoubtedly represent a harm reduction in comparison to cigarettes [19]. However, E- cigarette usage still results in systemic exposure to numerous and potentially harmful vapour constituents following inhalation, including nicotine, flavouring chemicals and reactive aldehydes generated during vapourisation of humectants; propylene glycol and vegetable gylcerine, particularly to highly vascularised tissues such as the bone (Fig. 1) [20]. Critically, recent data suggests that vaping may be considerably more harmful that first thought. For example, exposure of human alveolar macrophages to E-cigarette vapour condensate resulted in increased inflammatory cytokine production, cell death and a reduced phagocytic ability [21]. Additionally, RNA sequencing of oral cavity epithelial cells identified significant dysregulation of cancer-associated genes in E-cigarette users, in comparison to control individuals [22]. Similarly, Tang et al. demonstrated the development of both lung adenocarcinomas and bladder urothelial hyperplasia in mice exposed to E-cigarette vapour over 54 weeks [23].

Given the established detrimental effects of smoking on bone health and the clinical implications for those undergoing orthopaedic surgery it is clearly important to understand the effects of E-cigarette smoking on bone. However, there has been limited investigation into the impact of E-cigarette usage on bone health, particularly over extended time periods and there are no clinical recommendations regarding E-cigarette usage either pre or post-orthopaedic surgery. In this literature review, we highlight studies that have investigated the impact of E-cigarette vapour and its major constituents on bone, detailing the models utilised and the mechanistic and functional results regarding bone health (Table 1).

**Table 1:** The impact of major E-cigarette vapour constituents on bone cell function

Constituent	Model	Treatment	Proliferation/viability	Gene Expression	ALP Activity	Bone Nodules	Other Cellular Functions	Ref
**Nicotine**	Primary human osteoblasts	3d, 0.01–50 mM	Up to 0.01 mM increased proliferation, > 1 mM reduced proliferation Reduced proliferation. Cytotoxic at 50 mM	↑Type I collagen, ostrix, ↓ALP, RUNX2, BSP, osteopontin, osteonectin	–	↑ with 1 mM	Altered morphology	[24]
	Primary human osteoblasts	0.01 μM-10 mM up to 3d	↑ Proliferation with doses up to 1 μM, ↓ with doses > 0.1 mM	↑ c-fos with 0.1 μM, 1 h	–	–	–	[25]
	Primary human osteoblasts	0.1 μM, 11 and 21d	Cytotoxic	–	–	–	↑ H_2_O_2_ accumulation, activation of caspase 3 and mitochondrial apoptosis pathways	[26]
	Murine cell line (OCCM.30)		–	↑ PGE2	↓		Time-dependent increase in nitric oxide production	
	Saos-2 cells	3 mM, up to 14d	–	OPG, PGE2, no change	↓	–	–	[27]
	MG63	0.01 μM- 10 mM 1d- 3d	0.01–100 μM increased proliferation, 1–10 mM decreased/cytotoxic	Type I Col, ALP, osteocalcin, ↑24 h 0.1 μM-1 mM ↓24 h 1-10 mM, 72 h all dosages	–	–	–	[28]
	RAW264.7 cells, Treated with RANKL for 7d	0.01–1 mM, up to 7d	–	↑ Carbonic anhydrase, α 7 nAch receptor ↓CatK, MMP-9, and V-ATPase d2	n/a	n/a	↓multinuclear osteoclasts with large nuclei	[29]
**Flavouring chemicals**	MG-63	Cinnamon flavoured, nicotine-free e-cigarette liquid and condensate, 2d.	↓ Viability	–	–	–	↑ in ROS production	[30]
	U937 and MM6 monocytic cell lines	Diacetyl, cinnamaldehyde, acetoin, maltol, pentanedione, o-vanillin, and coumarin, 0.01–1 mM	↓ Viability	–	–	–	↑ IL-8 cytokine secretion ↑ in ROS production	[31]
	Primary human bronchial epithelial (NHBE) cells	Diacetyl or 2,3-pentanedione, for 1d	–	RNA-seq differentially expressed genes: Diacetyl = 163 genes, 2,3-pentanedione = 568 genes	–	–	Disrupting cilia biogenesis	[32]
**Carbonyl compounds**	Human osteogenic sarcoma cell line (U2OS)	0.001–4 mM formaldehyde, 1-3d	↓ Proliferation, viability	–	–	–	–	[33]
	Human bone marrow stem cells cultured in osteogenic conditions	Acetaldehyde (0.1–0.12 mM) and Acrolein (0.01–0.12 mM) 1-28d	↓ Proliferation, viability	–	↓	↓ with 0.03 mM acrolein, 0.1 mmol/L acetaldehyde	Altered cell morphology Reduced adherence to titanium surface	[34]
	Mouse primary osteoblastic cells/ MC3T3-E1 murine cell line	0.04% Acetaldehyde, 1-4d	↓ Proliferation, viability	↑ PPARγ ↓ RUNX2, osterix	–	–	Reduced osteoblast differentiation, instead a shift towards adipogenesis	[35]
	Rat calvarial osteoblasts, bone marrow stromal cells	0.002% Acetaldehyde, 1-14d	–	↓ BMP-2, ALDH2	↓	↓	–	[36]

*RUNX2* Runt-related transcription factor 2, *BSP* Bone sialoprotein, *PGE2* Prostaglandin E2, *OPG* osteoprotegerin, *MMP* Matrix metalloproteinase, *ROS* reactive oxygen species, *PPAR-γ* Peroxisome proliferator-activated receptor gamma, *ALDH2* Aldehyde dehydrogenase 2, *Saos-2 / MG-63* - human osteoblast-like cell lines derived from patients with osteosarcoma. *RAW264.7* a monocyte/macrophage like cell linage, capable of forming multinucleated osteoclast-like cells), *ALP* Alkaline phosphatase, *CatK* Cathepsin K

## The impact of E-cigarette vapour on bone function in vivo

Currently, only one study has investigated the impact of E-cigarette vapour on bone in vivo*.* In this study, mice were exposed to E-cigarette vapour aerosol for 3 h per day, for up to 6 months [37]. No significant effect on cortical bone strength, bone stiffness or hydroxyapatite content was reported, however E-cigarette vapour did impact bone architecture, with microfractures evident in the femur of mice. Importantly, microfractures occurred in response to aerosols containing only propylene glycol and vegetable glycerol, suggesting this effect was not entirely mediated by nicotine or flavouring chemicals [37]. Notably, due to the investigation of other end points, atherosclerosis-prone apolipoprotein E-deficient (ApoE^−/−^) mice were utilised in this study [37]. Apolipoprotein E is a key protein involved in lipid transport. Consequently ApoE^−/−^ mice typically display increased atherosclerotic plaque development, in addition to systemic inflammation [38]. Subsequently these mice are often used to model atherosclerosis and also diseases of pulmonary inflammation such as chronic obstructive pulmonary disease [38]. However, ApoE^−/−^ mice have also previously been demonstrated to display increased bone mass compared to wild-type animals, due to increased bone formation mediated by osteoblasts [39]. Therefore, as mice used in this investigation may have had greater baseline bone mass compared to wild type mice, further study, ideally utilising human E-cigarette user cohorts is needed.

## Evidence for the impact of E-cigarette vapour and liquid on bone cell function

Rouabhia et al. investigated the impact of nicotine rich E-cigarette vapour on both osteoblast function (the main cell type responsible for the synthesis of new bone) and their ability to interact with dental implant disks [40]. Exposure of Saos-2 osteoblasts (human osteoblast-like cell line derived from a patient with primary osteosarcoma) to E-cigarette vapour resulted in reduced adherence of Saos-2 osteoblasts to the dental implant surface, potentially due to reduced expression of the adhesion molecule, F-actin [40]. Functionally, E-cigarettes caused a reduction in both mineralisation and alkaline phosphatase activity, a key enzyme in osteogenesis [40]. E-cigarette vapour exposure also upregulated expression of the pro-apoptotic gene caspase-3 and increased cell death. Notably, the detrimental effects of nicotine-rich E-cigarette vapour on Saos-2 osteoblast function were greater than nicotine-free E-cigarettes vapour, suggesting nicotine plays a significant role in the impact of E-cigarette vapour on Saos-2 osteoblast function [40].

In addition to E-cigarette vapour, A detrimental effect of commercially available E-liquids on osteoblast cell lines has also been reported. Direct application of a variety of E-liquids to human Saos-2 and MG-63 cells, (another osteoblast-like cell line, derived from a human osteosarcoma) at concentrations up to 4%, delivering a nicotine dose of up to 1 mg/ml (falling within a theoretical physiological exposure range of 1-3 mg of nicotine per cigarette) reduced cellular viability, independent of nicotine [41]. Interestingly, the response of the osteoblast cell lines to direct E-liquid exposure differed depending on flavour [41]. Although such direct application of E-cigarette liquid does not directly mimic real life usage, particularly as the chemical composition of E-cigarettes can change upon vaping [42–45], the results of these studies do highlight the potential harm E-cigarettes may have on bone health.

## The impact of nicotine on osteoblast cell lines

There is evidence that E-cigarette users self-titrate consumption to achieve a nicotine dose to which they were previously accustomed to when smoking cigarettes [46]. Therefore, the impact of nicotine on bone following the use of E-cigarettes may be comparable to that of cigarettes. Critically, the expression of acetylcholine receptor subunits has been reported in both human trabecular bone and primary human osteoblasts [25]. Additionally, α7 nicotinic receptor subunits are expressed in Saos-2 cells, with expression upregulated in response to nicotine [47]. Subsequently, a number of studies have investigated the effect of nicotine on bone function and phenotype utilising cell lines, human primary osteoblasts and human bone tissue [24, 26, 47, 48].

A nicotine-mediated dose dependent decrease in proliferation was observed in Saos-2 cells for up to 14 days in culture [46]. Critically, nicotine (10 mM, up to 14 days) also reduced the formation of bone-like nodules, structures formed by the mineralisation of the extracellular matrix secreted by osteoblasts [48]. In support of this, Alkaline phosphatase activity and type I collagen expression in Saos-2 cells was also significantly reduced with nicotine treatment [48]. Furthermore, both mRNA and protein expression of matrix metalloproteinases (MMP) -1, 2, 3 and 13 has been reported to be significantly greater in Saos-2 cells in response to nicotine treatment for 5–10 days [47]. In contrast, no effect on the expression of tissue inhibitors of metalloproteinases (TIMPS) was observed. MMPs degrade extracellular matrix proteins such as collagens, elastin and glycoprotein, which in this context include bone extracellular matrix proteins such as type I collagen [49]. An increased expression of MMPs with no corresponding increase in tissue TIMPs would therefore suggest increased extracellular matrix degradation [47].

Nicotine has also been implicated as a driving force of mitochondrial stress and reactive oxygen species (ROS) production in osteoblast-like cells differentiated from mouse mesenchymal stem cells. In this model, nicotine reduced sirtuin-3 (sirt3 expression), subsequently inhibiting mitochondrial anti-oxidative enzymes, while a time-dependent increase in nitric oxide production has been reported in a murine immortalized cementoblast cell line (OCCM.30) [50, 51].

## The impact of nicotine on human primary osteoblasts

A bimodal effect of nicotine on primary human osteoblast proliferation was demonstrated by Walker et al. In this study, low doses of nicotine (0.01–10 μM for 72 h) promoted osteoblast proliferation, potentially mediated via an induction of c-fos oncogene. In contrast, higher doses (> 1 mM for 72 h) reduced osteoblast proliferation and caused cell death [25]. Interestingly, nicotine infusion of human trabecular bone tissue increased osteopontin protein expression. Since osteopontin is implicated in bone resorption, increased osteopontin expression may be indicative of a progressive loss of bone mass in vivo [25]. Marinucci et al. performed similar experiments, in which primary human osteoblasts were exposed to nicotine for 11 and 21 days. Application of the lowest dose of nicotine (0.1 μM) significantly reduced osteoblast viability, with increased activation of caspase-3. Nicotine also induced osteoblast apoptosis by both increasing the accumulation of H_2_O_2_ and inhibiting nuclear factor kappa-light-chain-enhancer of activated B cells (NF-KB) activation, as evidenced by reduced nuclear levels of NF-kB p65, which subsequently resulted in upregulation of B-cell lymphoma 2 (Bcl-2), bcl-2-associated X protein (BAX) and caspase-3 and in-turn apoptosis [26]. In a separate study, Marinucci et al. also demonstrated increased type I collagen mRNA expression in primary human osteoblasts in response to acute nicotine stimulation, whilst alkaline phosphatase mRNA expression decreased [24]. Chronic treatment of human osteoblasts with nicotine induced a downregulation in the expression of the osteoblast master transcription factor runt-related transcription factor 2 (RUNX2), indicating inhibition of osteoblast differentiation. In support of this, reduced expression of mRNAs coding for bone matrix proteins such as bone sialoprotein (BSP), osteopontin, and osteonectin were also observed with chronic nicotine exposure [24].

## The impact of nicotine on osteoclast function

Osteoclasts are primarily responsible for bone resorption and therefore a positive balance between osteoclast and osteoblast activity leads to a reduction in bone mass [52]. Similarly to osteoblasts, osteoclasts express α1–5, 7, 9 and 10 nicotine receptor subtypes, with α7 mRNA increasing in response to nicotine treatment, in a dose dependent fashion [29]. In vitro studies investigating the effect of nicotine on osteoclast function have reported differing results. On one hand, nicotine increases carbonic anhydrase expression, an enzyme necessary for the generation of H^+^ ions, that when accumulated extracellularly, promote demineralisation [29]. On the other hand, nicotine treatment decreased V-ATPase expression. V-ATPase is responsible for the export of H^+^ ions from osteoclasts, suggesting reduced bone resorption [53]. In support of the latter, nicotine has also been demonstrated to reduce the planar area of the resorption in RAW264.7 cells (a monocyte/macrophage like cell linage, capable of forming multinucleated osteoclast-like cells) [29]. Similarly conflicting results have been observed in vivo. Knockout of α7 nicotine receptors in mice resulted in decreased osteoclastogenesis, while circulating osteoprotegerin (OPG) was elevated, resulting in increased bone mass [54, 55]. However in contrast, Mito et al. demonstrated that nicotine mediated activation of α7 receptors in mice promoted the upregulation of receptor activator of nuclear factor kappa-Β ligand (RANKL) and inhibited osteoprotegerin OPG expression, consequently promoting osteoclast activation and bone resorption [55].

## The impact of E-cigarette flavouring chemicals on bone health

The wide variety of E-cigarette flavouring liquids available for consumption (over 8000 to date) is a primary contributing factor to the rise in popularity of E-cigarette usage, especially amongst younger individuals and non-smokers [56–58]. However, there is limited regulation and quality control of flavouring compounds present in E-cigarette liquids and there is a dearth of studies investigating their safety and physiological effects on bone.

Evidence from the monocytic cell lines U937 and MM6 demonstrated that flavouring chemicals can evoke a number of direct harmful effects, including reduced cell viability and increasing pro-inflammatory cytokine production [58]. Similarly, exposure of primary normal human bronchial epithelial (NHBE) cells to two commonly used flavouring chemicals (diacetyl or 2,3-pentanedione) for 24 h evoked differential expression of 163 and 568 genes respectively [32], while a cytotoxic effect on mouse neuronal cells has also been reported [59]. Additionally, there is evidence that cinnamaldehyde supresses innate immune cells function, reducing macrophage and neutrophil phagocytosis, neutrophil extracellular trap formation and natural killer cell cytotoxicity [60]. Regarding bone, Wavreil and Heggland investigated the effect of cinnamon flavouring on MG-63 cell function indirectly, by comparing cinnamon flavoured nicotine free E-cigarette liquid and condensate against unflavoured controls. 48 h exposure to flavoured liquid or condensate significantly reduced MG-63 viability, likely attributable to oxidative stress [30].

Although lower than reported in cigarette smoke (> 10^16^ molecules/puff), upon vaporising, E-cigarette liquids generate a considerable amount of short lived, highly reactive free radicals (> 10^13^ molecules/puff) [42, 45, 61, 62]. Additionally, atomization of flavouring chemicals including linalool, dipentene, and citral also caused free radical production [63]. Considering that ROS are associated with osteoclast activation and bone resorption, flavouring agent derived free radicals may therefore negatively impact on bone [64].

Collectively these data highlight the need to determine the level of exposure of flavouring chemicals in bone and the potential harmful effects flavouring chemicals may have in humans, particularly with chronic repeated exposure.

## The impact of E-cigarette derived glycols on bone health

Most E-cigarette liquids contain either a glycol such as propylene glycol or glycerine. Such compounds are necessary to form vapour central to E-cigarette usage in addition to acting as solvents, facilitating the addition of flavouring chemicals. Such compounds have generally been considered safe due to their approval for oral consumption in food products [65]. Critically, such safety recommendations do not encompass their inhalation upon combustion, as individuals are exposed to when vaping. As a result, potentially harmful effects, especially with regard to bone have currently been overlooked.

## The impact of E-cigarette derived carbonyl compounds on bone health

The most common carrier agents/humectants used in E-cigarette liquids include propylene glycol and vegetable glycerine. Thermal degradation of these compounds occurs during the use of E-cigarettes, generating carbonyl compounds such as formaldehyde, acetaldehyde and acrolein [42, 66]. Importantly, increased amounts of these aldehyde compounds were detected in exhaled breath following vaping, with very high (95%) uptake by the respiratory tract also demonstrated [67]. Critically, the amounts of formaldehyde exhaled were reportedly similar to traditional cigarettes (~ 5 μg·puff− 1) [67].

Although the effect of E-cigarette derived carbonyl compounds on bone cell function has not been studied directly, formaldehyde and acetaldehyde have been demonstrated to reduce proliferation and increased cell death of U2OS cells (a human osteoblastic cell line) in a dose dependent manner [33, 68]. Acetaldehyde and Acrolein also inhibited osteoblast alkaline phosphatase activity and mineralisation [34]. Importantly, this study also identified that aldehyde treatment could inhibit osteoblasts adherence to an implant surface and such effects are independent of nicotine [34].

Acetaldehyde has also been shown to stimulate PPARγ expression in mouse osteoblast cells, a transcription factor that inhibits osteoblast differentiation [35]. Additionally, mice expressing a dominant-negative form of aldehyde dehydrogenase 2 (ALDH2), an enzyme which catalyses the conversion of acetaldehyde to acetic acid, exhibit an osteoporotic phenotype [35]. In support of this, increasing ALDH2 activity, promoted alkaline phosphatase activity and bone nodule formation in primary rat osteoblasts [36]. In human individuals, genetic polymorphisms resulting in reduced ALDH2 cause a build-up of acetaldehyde, lower BMD and a significantly increased rate of hip fracture and osteoporosis [69, 70].

Collectively, these data suggest that increased exposure of bone to carbonyl compounds following E-cigarette usage may negatively affect osteoblast function. However, studies investigating the impact of carbonyl compounds on both human primary osteoblast and osteoclast function are required. There is also a need to determine the amounts of carbonyl compounds exposure when vaping and the impact of chronic exposure.

## The impact of E-cigarette derived metal particulates on bone health

Traditional cigarettes are associated with the inhalation of metal particulates, including chromium, cadmium, lead and nickel which are known to have a variety of harmful effects [71–73]. Emerging data has demonstrated that metal particulates and nanoparticles are also generated upon the use of E-cigarettes, with concentrations in many cases similar or exceeding those from cigarette smoke [74, 75]. The effect of E-cigarette derived metal particulates on bone has not yet been considered, however the impact of various metal particulates on bone have been reported in other scenarios, recently reviewed by Rodríguez and Mandalunis [76]. Sustained exposure to E-cigarette derived cadmium may be of particular importance to bone health, as increased accumulation in smokers is associated with bone resorption, demineralisation and increased risk of osteoporosis and fracture [77–80]. Therefore, although cadmium absorption is reportedly lower in E-cigarette users compared to smokers [81], it is important to elucidate the impact of chronic exposure to cadmium and other metal particulates on bone function in humans following E-cigarette use.

## The impact of E-cigarette vapour on the osteogenic differentiation of mesenchymal stem cells

In addition to osteoblasts and osteoclasts discussed above, it is important to consider the impact of E-cigarette usage on mesenchymal stem cells (MSCs). Bone marrow is a major source of MSCs and their differentiation towards the osteoblast lineage is involved in the regulation of bone turnover, particularly following injury [82]. MSCs cultured in osteogenic conditions displayed reduced expression of alkaline phosphatase mRNA, significantly reduced type I collagen (COL1) mRNA expression and a reduction in mineralisation following treatment with E-cigarette smoke extract [83]. The authors also report a striking reduction in connexin43 protein expression [83]. Connexin43 facilitates gap junction formation and their presence has been suggested to play a role in osteogenic differentiation of MSCs [84]. Therefore, loss of connexin43 may offer a possible mechanism for E-cigarette mediated inhibition of MSC commitment to the osteoblast lineage.

## The impact of Cannabidiol (CBD) on bone health

The development of E-cigarette devices has also resulted in their utilisation to vape cannabidiol (CBD), the major non-psychoactive constituent of cannabis, due to its purported analgesic, anti-inflammatory, and anti-epileptic properties [85–87]. When examining the direct effect of CBD on bone, Napimoga et al. demonstrated that 5 mg/KG/day CBD reduced bone loss following the induction of periodontal disease in rats, potentially by reducing osteoblast RANKL production [88]. Similarly, CBD promoted bone healing in rats following femoral fracture, possibly mediated by increased collagen crosslinking though increased, lysyl hydroxylase 1 (PLOD1) expression, a collagen crosslinking enzyme [89]. In addition, intradiscal injection of CBD prevented intervertebral disk degeneration in rats, following injury [90].. Most recently, Li et al. demonstrated that intradiscal CBD treatment decreased RANKL and increased OPG mRNA expression in rats following complete spinal transection [91]. Continued treatment with CBD for 2 weeks following spinal injury also increased bone volume and trabecular thickness in these animals [91]. There is also evidence that CBD can supress osteoclastogenesis, and reduce the function of human osteoclasts by acting as a G protein-coupled receptor 55 (GRP55) receptor antagonist, further supporting a beneficial effect of CBD on bone formation [92, 93]. Together, these data appear to support a positive effect of CBD on bone, although critically, studies to examine the impact of CBD obtained via E-cigarette use on human bone is required. Indeed, it will be important to consider whether delivery of CBD through the medium of an E-cigarette is a credible option to facilitate bone healing, especially considering both the poor regulation and quality control surrounding such products and the considerable harm the user may be inflicting to bone via other constituents within the CBD liquid, as discussed above, in addition to other organs such as the lungs particularly with prolonged use [94, 95]. Consequently any potential benefit to bone is likely to be outweighed by such harmful effects.

## Conclusions

E-cigarette usage is commonly regarded as a safe alternative to smoking cigarettes. However, there is evidence that many of the major constituents of E-cigarettes, such as nicotine and carbonyl compounds can significantly impair osteoblast function, suggesting E-cigarette use may be detrimental to bone health. Future studies are clearly needed to investigate both the long-term effect of E-cigarette usage on bone function in humans and their potential impact on bone associated disease, injury and orthopaedic surgery. Subsequently this will inform users of potential health risks and may help to increase recovery and limit complications following orthopaedic surgery.

## Data Availability

Not applicable.

## Abbreviations

ApoE: atherosclerosis-prone apolipoprotein E-deficient
ALDH2: Aldehyde dehydrogenase 2
Bax: bcl-2-associated X protein
Bcl-2: B-cell lymphoma *2*
BMD: Bone mineral density
BMI: Body mass index
BSP: Bone sialoprotein
CBD: cannabidiol
COL1: Type 1 collagen
E-cig: electronic cigarette
GRP55: G protein-coupled receptor 55
MMP: Matrix metalloproteinase
NF-κB: nuclear factor kappa-light-chain-enhancer of activated B cells
NHBE: normal human bronchial epithelial
OPG: osteoprotegerin
PGE2: Prostaglandin E2
RANK-L: Receptor activator of nuclear factor kappa – beta
ROS: reactive oxygen species
RUNX2: Runt-related transcription factor 2
TIMP: Tissue inhibitors of metalloproteinase
